# Supplementary material for: Novel Antimicrobial Peptides from a Cecropin-Like Region of Heteroscorpine-1 from Heterometrus laoticus Venom with Membrane Disruption Activity
Source: Molecules. 2021 Sep 28;26(19):5872. doi: 10.3390/molecules26195872 (PMC8512776; doi:10.3390/molecules26195872)
Supplement: Supplementary file 1 [file molecules-26-05872-s001.zip › Supplement 1 HPLC analysis CeHS-1.pdf]

Sample Name : CeHS-1
Sample ID :U461AFJ270-1
Time Processed: 3:51:25
Year-Month-Day: 2020/12/16

Pump A : 0.065% trifluoroacetic in 100% water (v/v)
Pump B : 0.05% trifluoroacetic in 100% acetonitrile (v/v)
Total Flow:1 ml/min
<<Detector A>>
Wavelength Ch1 : 220 nm

<<LC Time Program>>

Time	Module	Command	Value	Comment
0.01	Pumps	Solvent B Conc.	5	
25.00	Pumps	Solvent B Conc.	65	
25.01	Pumps	Solvent B Conc.	95	
27.00	Pumps	Solvent B Conc.	95	
27.01	Pumps	Solvent B Conc.	5	
33.00	Pumps	Solvent B Conc.	5	
33.01	Controller	Stop		

<<Column Performance>>
<Detector A>
Column : Inertsil ODS-3 4.6 x 250 mm
Equipment:ZJ19010015

<Chromatogram>

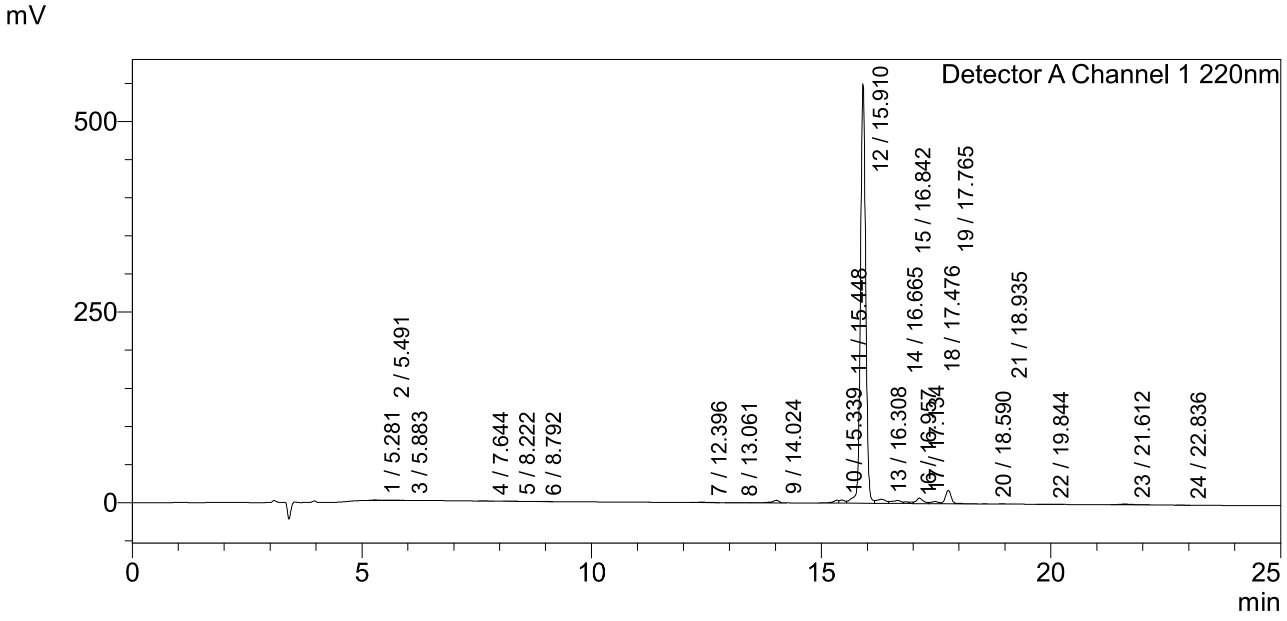

<Peak Table>

Detector A Channel 1 220nm

Peak#	Ret. Time	Area	Height	Area%
1	5.281	9725	898	0.197
2	5.491	12762	551	0.258
3	5.883	4534	331	0.092
4	7.644	2404	249	0.049
5	8.222	1625	141	0.033
6	8.792	1978	117	0.040
7	12.396	9032	550	0.183
8	13.061	3022	248	0.061
9	14.024	39773	3359	0.804
10	15.339	36871	3815	0.745
11	15.448	34451	4134	0.696
12	15.910	4328726	550172	87.504
13	16.308	76579	5397	1.548
14	16.665	56302	4015	1.138

Peak#	Ret. Time	Area	Height	Area%
15	16.842	15393	2203	0.311
16	16.957	11235	2077	0.227
17	17.134	76893	7255	1.554
18	17.476	32135	2847	0.650
19	17.765	158729	17626	3.209
20	18.590	1419	156	0.029
21	18.935	5299	506	0.107
22	19.844	3164	169	0.064
23	21.612	23321	1000	0.471
24	22.836	1518	203	0.031
Total		4946891	608020	100.000
