# Supplementary material for: Novel Antimicrobial Peptides from a Cecropin-Like Region of Heteroscorpine-1 from Heterometrus laoticus Venom with Membrane Disruption Activity
Source: Molecules. 2021 Sep 28;26(19):5872. doi: 10.3390/molecules26195872 (PMC8512776; doi:10.3390/molecules26195872)
Supplement: Supplementary file 1 [file molecules-26-05872-s001.zip › Supplement 2 HPLC analysis CeHS-1 GP.pdf]

Sample Name :CeHS-1 GP
Sample ID :U461AFJ270-3
Time Processed :10:39:10 AM
Month-Day-Year Processed :12/12/2020

Pump A : 0.065% trifluoroacetic in 100% water (v/v)
Pump B : 0.05% trifluoroacetic in 100% acetonitrile (v/v)
Total Flow:1 ml/min
Wavelength:220 nm
<<LC Time Program>>

| Time | Module | Command | Value |
|---|---|---|---|
| 0.01 | Pumps | B.Conc | 5 |
| 25.00 | Pumps | B.Conc | 65 |
| 25.01 | Pumps | B.Conc | 95 |
| 27.00 | Pumps | B.Conc | 95 |
| 27.01 | Pumps | B.Conc | 5 |
| 35.00 | Pumps | B.Conc | 5 |
| 35.01 | Controller | Stop | |

<<Column Performance>>
<Detector A>
Column :Inertsil ODS-3 4.6 x 250 mm
Equipment: ZJ17010508

## <Chromatogram>

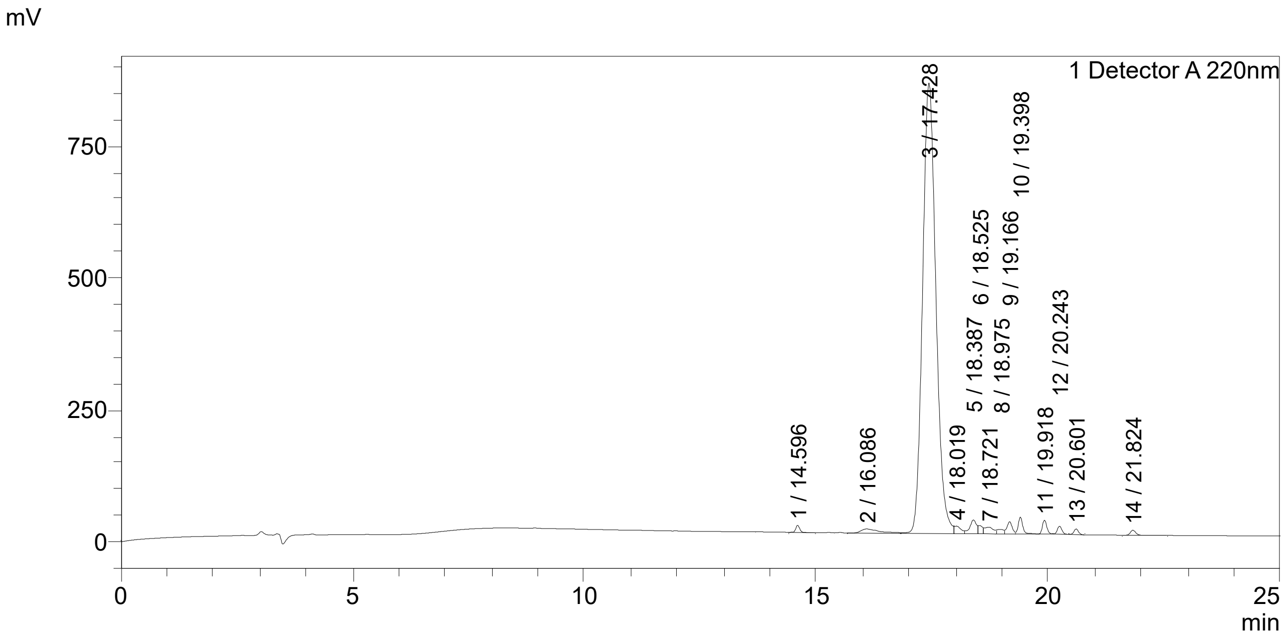


## <Peak Table>

Detector A 220nm

| Peak# | Ret. Time | Area | Height | Area% |
|---|---|---|---|---|
| 1 | 14.596 | 81360 | 13610 | 0.431 |
| 2 | 16.086 | 227719 | 8021 | 1.205 |
| 3 | 17.428 | 16994249 | 853880 | 89.925 |
| 4 | 18.019 | 136979 | 14281 | 0.725 |
| 5 | 18.387 | 270894 | 26305 | 1.433 |
| 6 | 18.525 | 109452 | 15655 | 0.579 |
| 7 | 18.721 | 148978 | 12711 | 0.788 |
| 8 | 18.975 | 94597 | 8467 | 0.501 |
| 9 | 19.166 | 184083 | 23036 | 0.974 |
| 10 | 19.398 | 224557 | 32004 | 1.188 |
| 11 | 19.918 | 178056 | 26440 | 0.942 |
| 12 | 20.243 | 102117 | 15164 | 0.540 |
| 13 | 20.601 | 67839 | 10531 | 0.359 |
| 14 | 21.824 | 77417 | 9418 | 0.410 |
| Total | | 18898297 | 1069523 | 100.000 |
