# Supplementary material for: Novel Antimicrobial Peptides from a Cecropin-Like Region of Heteroscorpine-1 from Heterometrus laoticus Venom with Membrane Disruption Activity
Source: Molecules. 2021 Sep 28;26(19):5872. doi: 10.3390/molecules26195872 (PMC8512776; doi:10.3390/molecules26195872)
Supplement: Supplementary file 1 [file molecules-26-05872-s001.zip › Supplement 3 HPLC analysis CeHS-1 GPK.pdf]

Sample Name :CeHS-1 GPK
Sample ID :U461AFJ270-5
Time Processed :12:51:01 AM
Month-Day-Year Processed :12/09/2020

Pump A : 0.065% trifluoroacetic in 100% water (v/v)
Pump B : 0.05% trifluoroacetic in 100% acetonitrile (v/v)
Total Flow:1 ml/min
Wavelength:220 nm
<<LC Time Program>>

Time	Module	Command	Value
0.01	Pumps	Pump A B.Conc	5
25.00	Pumps	Pump A B.Conc	65
25.01	Pumps	Pump A B.Conc	95
27.00	Pumps	Pump A B.Conc	95
27.01	Pumps	Pump A B.Conc	5
35.00	Pumps	Pump A B.Conc	5
35.01	Controller	Stop	

<<Column Performance>>
<Detector A>
Column :Inertsil ODS-3 4.6 x 250 mm
Equipment: GK11010009

<Chromatogram>

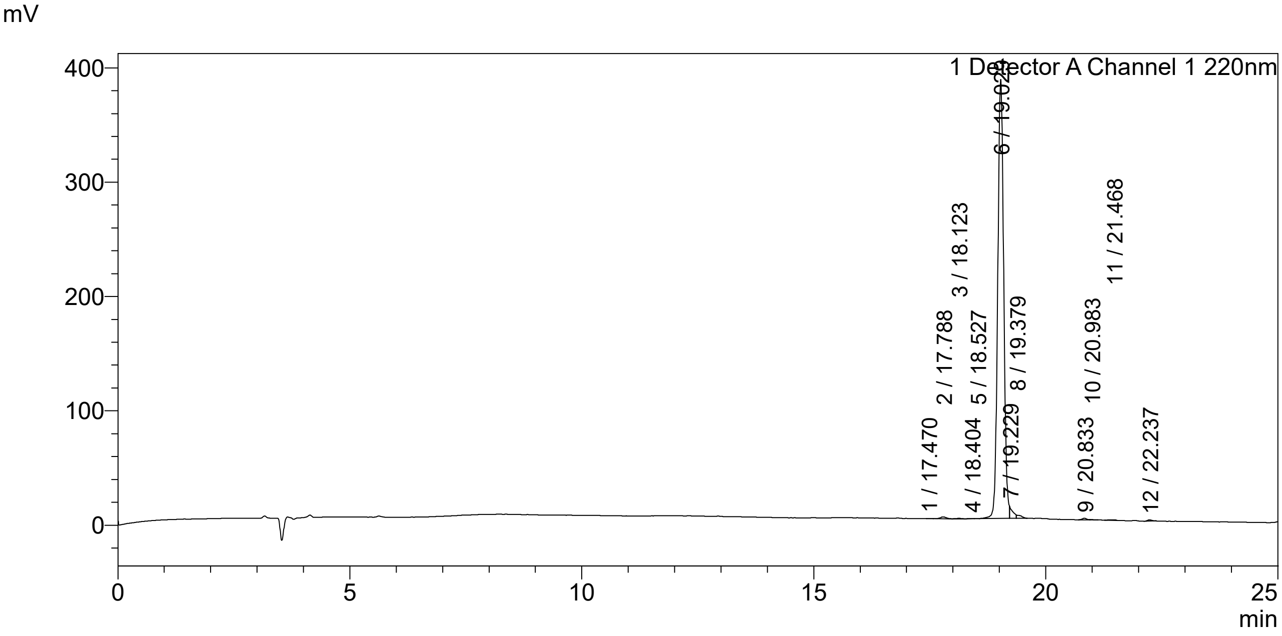

<Peak Table>

Detector A Channel 1 220nm

Peak#	Ret. Time	Area	Height	Area%
1	17.470	1050	171	0.030
2	17.788	15007	1678	0.423
3	18.123	3102	429	0.088
4	18.404	926	162	0.026
5	18.527	900	144	0.025
6	19.029	3426331	384111	96.679
7	19.229	52383	9799	1.478
8	19.379	20299	2809	0.573
9	20.833	9963	1448	0.281
10	20.983	1641	293	0.046
11	21.468	5435	571	0.153
12	22.237	6990	1041	0.197
Total		3544027	402657	100.000
