# Supplementary material for: Novel Antimicrobial Peptides from a Cecropin-Like Region of Heteroscorpine-1 from Heterometrus laoticus Venom with Membrane Disruption Activity
Source: Molecules. 2021 Sep 28;26(19):5872. doi: 10.3390/molecules26195872 (PMC8512776; doi:10.3390/molecules26195872)
Supplement: Supplementary file 1 [file molecules-26-05872-s001.zip › Supplement 5 MS analysis CeHS-1 GP.pdf]

Mass Spectrum

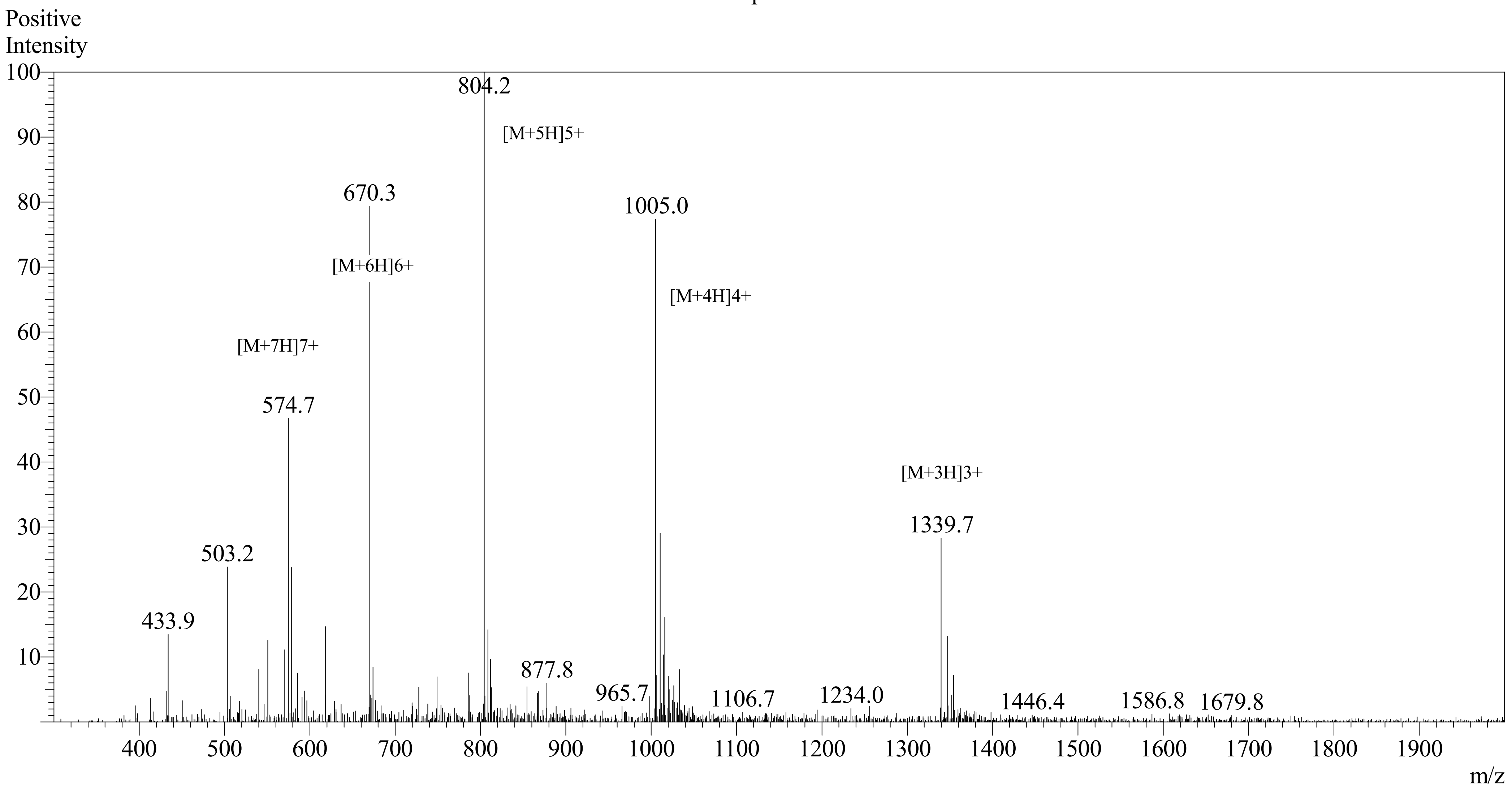

Sample Information	
Month-Day Processed :	12/10/20
Time Processed :	07:39:09 PM
Injection Volume :	0.3
Sample Name :	CeHS-1 GP
Sample ID :	U461AFJ270-3
Theoretical MW :	4016.78
Observed MW :	4016.0

Interface	:ESI
Nebulizing Gas Flow	:1.5L/min
CDL Temp	:250
Block Temp	:200

Equipment	: GK11010007
Interface Bias	: +4.5 kV
Drying Gas Flow	:5 L/min
T.Flow	:0.2 ml/min
B.conc	:50%H2O/50%MeOH
